# Heparanase promotes endothelial-to-mesenchymal transition in diabetic glomerular endothelial cells through mediating ERK signaling

**DOI:** 10.1038/s41420-022-00858-0

**Published:** 2022-02-16

**Authors:** Kaili Chang, Qiyuan Xie, Jianying Niu, Yong Gu, Zhonghua Zhao, Fengxia Li, Qiaojing Qin, Xueguang Liu

**Affiliations:** 1 Department of Pathology, School of Basic Medical Sciences, Fudan University, 131 Dongan Road, 200032 Shanghai, China; 2 Department of Nephrology, Zhongshan Hospital, Fudan University, 180 Fenglin Road, 200032 Shanghai, China; 3 Department of Nephrology, The Fifth People’s Hospital, Fudan University, 801 Heqing Road, 200240 Shanghai, China; 4 Center of Community-Based Health Research, Fudan University, 801 Heqing Road, 200240 Shanghai, China; 5 Department of Nephrology, Huashan Hospital, Fudan University, 12 Middle Urumqi Road, 200040 Shanghai, China

**Keywords:** Experimental models of disease, Diabetic nephropathy

## Abstract

Glomerular endothelial cells (GEnCs) dysfunction occurs at the early stage of diabetic nephropathy (DN). One of its characteristics is endothelial-to-mesenchymal transition (EndMT). Heparanase (HPSE) is the only known mammalian endoglycosidase capable of degrading heparin sulfates and has a prominent role in DN pathogenesis. However, whether HPSE induces EndMT of GEnCs remains unknown. This study aimed to determine the effect and potential mechanism of HPSE on GEnCs phenotype under high*-*glucose conditions. In the early development of streptozotocin (STZ)-induced diabetic mice, HPSE overexpression was positively correlated with renal injury and the number of GEnCs undergoing EndMT, which was characterized by loss of endothelial marker CD31 and gain of mesenchymal markers including α-SMA and Snail1/2 by double immunofluorescence staining. Bioinformatics analysis revealed a positive correlation between HPSE and ERK. The counts of double positive staining of CD31 and p-ERK1/2 was significantly increased in the glomeruli of STZ-induced diabetic mice compared with sham mice. In cultured GEnCs, high glucose dramatically upregulated the expressions of HPSE and p-ERK1/2, both of which were markedly blocked by HPSE siRNA. Furthermore, recombinant mouse HPSE (rmHPSE) promoted the expressions of mesenchymal markers and p-ERK1/2 in a dosage- and time-dependent manner. U0126, a specific MEK/ERK inhibitor, significantly inhibited either high glucose or rmHPSE-induced EndMT of GEnCs. These data indicate that high glucose induces EndMT of GEnCs at least partially through upregulating HPSE and that HPSE promotes EndMT of GEnCs via activating ERK signaling. This study improves understanding the crucial role of HPSE in DN development and progression.

## Introduction

Diabetes mellitus is a group of metabolic disorders characterized by hyperglycemia. The kidneys are prime target organs and diabetic nephropathy (DN) is one of the most common complications in patients with diabetes. DN can eventually progress to end*-*stage kidney disease and has become the leading cause of renal failure worldwide [1, 2]. However, the pathogenesis of DN have remained unclear.

DN mainly manifests as impaired glomerular filtration capacity. Glomerular filtration barrier (GFB) is composed of three layers: fenestrated endothelial cells, glomerular basement membrane (GBM) and podocytes, which restricts passage of proteins and macromolecules based on their size and charge. The damage of any layer of GFB increases glomerular permeability and results in proteinuria [3]. Glomerular endothelial cells (GEnCs) cover the luminal surface of glomerular capillaries and are the first layer of GFB to be exposed to circulating factors. GEnCs are covered with the glycocalyx, which is a gel-like layer consisting of glycoproteins and proteoglycans with bound glycosaminoglycans (GAGs) [4]. The highly negatively charged proteoglycans with GAG, in particular heparan sulfates (HS), are the main contributors to the glomerular charge barrier. The only known mammalian endoglycosidase capable of degrading HS is heparanase (HPSE) [5]. Its enzymatic degradation of HS results in a compromised glomerular endothelial glycocalyx and elevated albumin excretion [1, 5–7]. In addition, HPSE has been shown to play several important non-enzymatic roles in kidney diseases and renal fibrosis [5–7]. Increased expression of glomerular HPSE was observed in several kidney diseases. Its Knock-Out prevented the development of albuminuria in streptozotocin (STZ)-induced diabetic mice [8] and acute experimental glomerulonephritis [9]. Therefore, HPSE may serve as a therapeutic target for glomerular diseases.

GEnCs are the coordinator in DN development and play a crucial role in the progress of DN [10]. GEnCs dysfunction occurs in early stages of DN, and may precede and contribute to podocyte injury and mesangial expansion [3, 10]. Under pathologic conditions, injured GEnCs may undergo endothelial-to-mesenchymal transition (EndMT) [3, 5, 11]. EndMT is characterized by loss of the expression of endothelial markers such as CD31 and VE-cadherin, and acquisition of the expression of mesenchymal markers such as α-smooth muscle actin (α-SMA) and Snail [3, 5, 11]. In high glucose-treated GEnCs [12, 13] and in renal cortex tissues of diabetic rats [12], the protein levels of the mesenchymal markers increased while those of the endothelial markers declined. Similar changes were observed by immunostaining in the glomeruli of experimental diabetic mice [10, 14–16] and the patients with DN [17]. HPSE was previously shown to enhance the expression of mesenchymal markers in vascular endothelial cells of multiple myeloma and thus promoted myeloma progression [18]. However, the relationship between HPSE and EndMT of GEnCs has not been reported yet. Therefore, this study was conducted to explore the effect of HPSE on glomerular EndMT either in high glucose-treated GEnCs in vitro or in STZ-induced diabetic mice in vivo. This research may enrich the mechanisms of HPSE in DN pathogenesis and furthermore provides clues for DN treatment.

## Results

### HPSE had a positive correlation with proteinuria and glomerular mesangial matrix expansion in STZ-induced diabetic mice

**Fig. 1:**
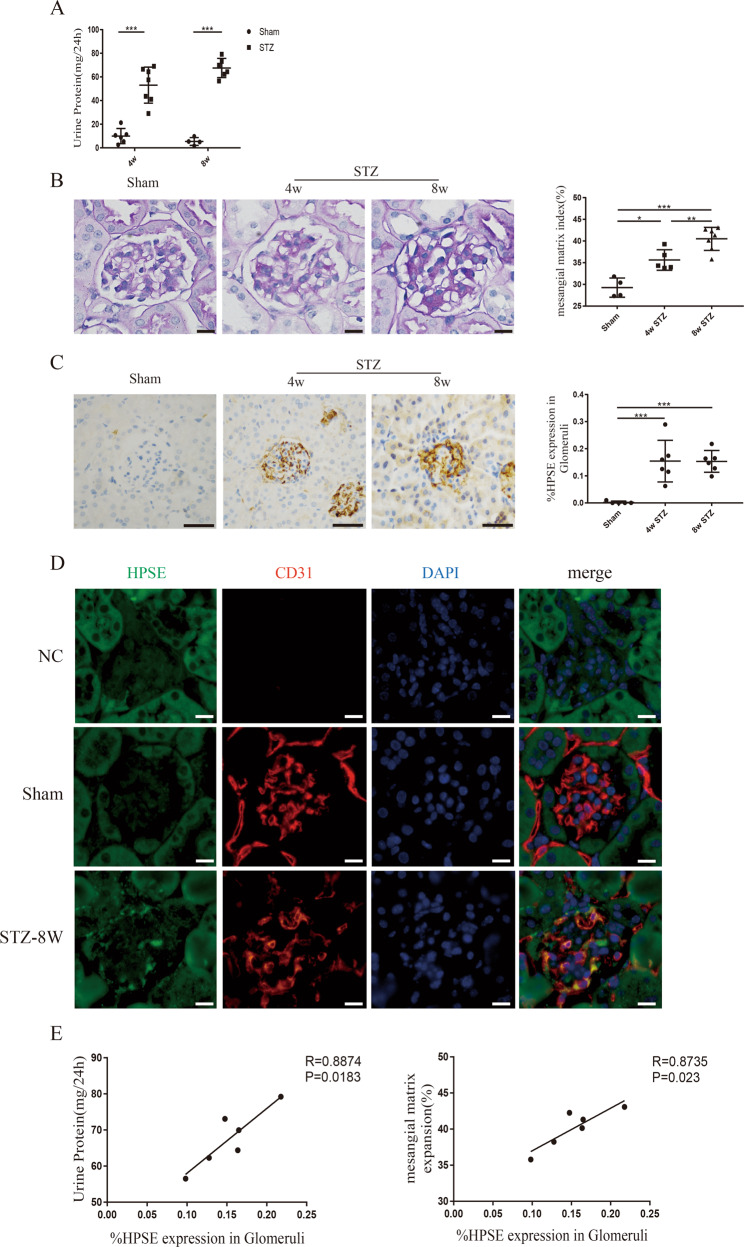
HPSE expression was upregulated in GEnCs of STZ-induced diabetic mice. **A** Quantitation of 24 h urine protein showed significant proteinuria at 4w and 8w respectively after the onset of diabetes in STZ-induced diabetic mice. **B** PAS staining exhibited dramatic mesangial matrix expansion in STZ-induced diabetic mice. Scale bar = 10 μm. **C** Immunohistochemical staining revealed the overexpressed HPSE in the glomeruli of diabetic mice. Scale bar = 50 μm. **D** The count of double positive immunofluorescence staining for CD31 and HPSE was markedly high in diabetic mice, while it was negative in sham animals. Scale bar = 10 μm. **E** HPSE expression was positively correlated with 24 h proteinuria (*R* = 0.8874, *P* = 0.0183) and glomerular mesangial matrix expansion (*R* = 0.8735, *P* = 0.023) respectively by Pearson correlation analysis. Each vertical bar represents the mean ± SD (*n* = 4~7) analyzed by one-way ANOVA or two-way ANOVA followed by Tukey’s multiple comparisons test. *P < 0.05, **P < 0.01, and ***P < 0.001.

Low-dose STZ-induced diabetic DBA/2 J mice exhibited massive proteinuria after the onset of diabetes. 24 h urinary protein quantification was 57.31 ± 17.46 mg at 4 weeks and 60.91 ± 19.95 mg at 8 weeks, respectively, which were significantly increased compared with sham mice (Fig. 1A). PAS staining revealed dramatic glomerular mesangial matrix expansion in diabetic kidney tissue (Fig. 1B). In addition, IHC staining showed that the expression of glomerular HPSE was clearly strong in diabetic mice while it was undetectable in nondiabetic animals (Fig. 1C). There was no significant difference of HPSE expression between 4 w and 8 w groups. Double positive IF staining of HPSE and the endothelial marker CD31 indicated a marked upregulation of HPSE expression in GEnCs of diabetic mice (Fig. 1D). Pearson correlation analysis showed a positive correlation between glomerular HPSE and 24 h proteinuria (*R* = 0.8874, *P* = 0.0183; Fig. 1E) or glomerular mesangial matrix expansion (*R* = 0.8735, *P* = 0.023; Fig. 1F), respectively.

### HPSE was positively associated with EndMT of GEnCs in STZ-induced diabetic mice

**Fig. 2:**
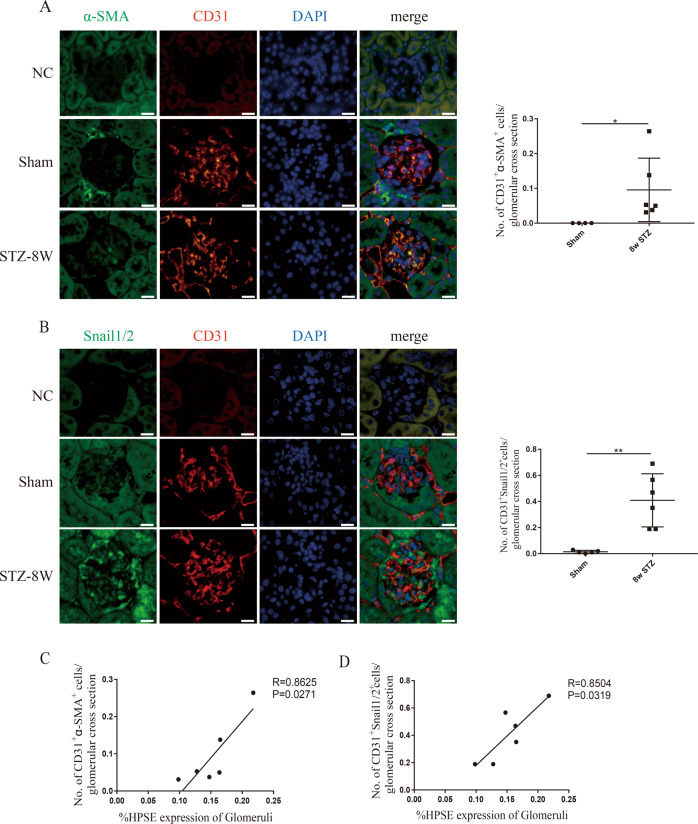
HPSE had a positive correlation with EndMT of GEnCs in STZ-induced diabetic mice. The counts of double positive IF staining for CD31-α-SMA (**A**) and CD31-Snail1/2 (**B**) were markedly high in STZ-induced diabetic mice. Pearson correlation analysis exhibited that HPSE was positively correlated with the numbers of double positive staining for CD31-α-SMA (*R* = 0.8625, *P* = 0.0271) (**C**) or CD31-Snail1/2 (*R* = 0.8504, *P* = 0.0319) (**D**) respectively. Scale bar = 10 μm. Each vertical bar represents the mean ± SD (*n* = 4~6) analyzed by two-tailed *t*-tests test. *P < 0.05, **P < 0.01, and ***P < 0.001.

The in situ recognition of EndMT in tissues depends on detection of cells expressing both mesenchymal and endothelial markers [11]. We performed double IF staining for the endothelial marker CD31 and the mesenchymal markers α-SMA (Fig. 2A) or Snail1/2 (Fig. 2B) to label EndMT. The double positive staining of CD31-α-SMA and CD31-Snail1/2 in glomeruli indicated GEnCs undergoing EndMT in diabetic mice, while it was undetectable in sham mice (Fig. 2A, B), indicating EndMT of GEnCs under diabetic condition. Pearson correlation analysis showed a positive correlation between glomerular HPSE expression and the numbers of double positive staining of CD31 with α-SMA (*R* = 0.8625, *P* = 0.0271; Fig. 2C) or Snail1/2 (*R* = 0.8504, *P* = 0.0319; Fig. 2D) respectively. These data suggest that HPSE expression positively correlates with glomerular EndMT in DN.

### Bioinformatics analysis revealed a positive correlation between HPSE and ERK in DN patients

**Fig. 3:**
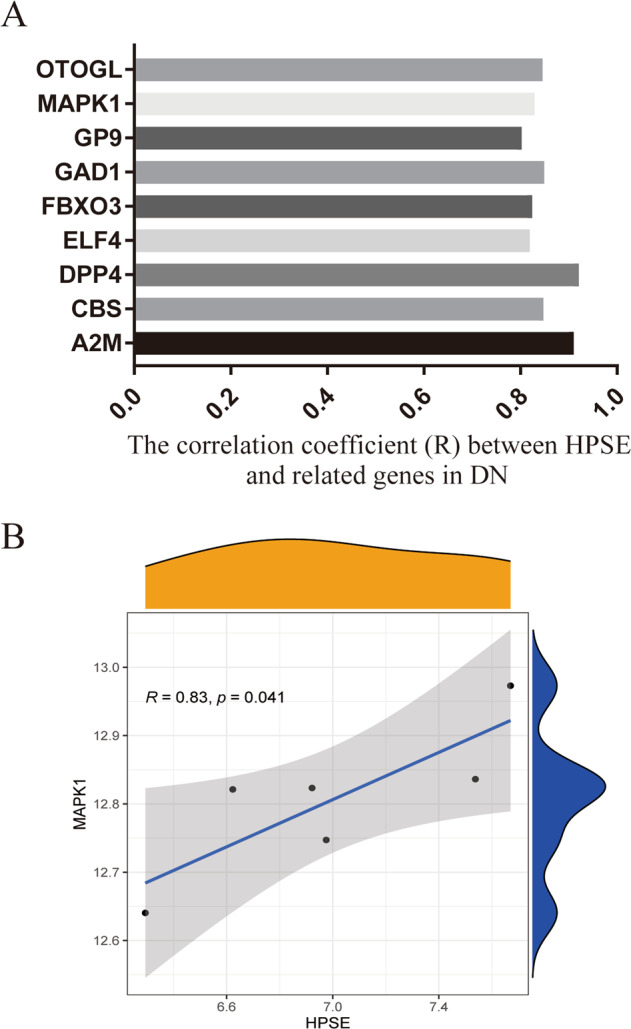
Bioinformatics analysis revealed a positive correlation between HPSE and ERK. The Pearson correlation analysis of DN patients’ data (*n* = 19) from GEO database (GSE14202) showed that nine related genes (**A**) had a significant positive correlation with HPSE, including MAPK1 (ERK2) (**B**) (*R* = 0.83, *P* = 0.041).

To explore the signaling pathway mediated by HPSE, we downloaded the gene expression profile data from the GEO database (GSE14202) of 19 cases of DN patients. Pearson correlation analysis showed that 20 related genes had a significant correlation with HPSE, and 9 of them were significantly positively related to HPSE (Fig. 3A). Among these nine related genes, only MAPK1 (i.e., ERK2) and DPP4 were reported to be involved in epithelial-mesenchymal transition (EMT) after reviewing the literature. Extracellular signal-regulated kinase (ERK) is a central molecule driving epithelial-mesenchymal transition (EMT) in cancer [19]. It has been reported to play an important role in DN development and progression [17, 20–22]. Pearson correlation analysis showed that HPSE and MAPK1 (i.e. ERK2) were significantly positively correlated (*R* = 0.83, *P* = 0.041; Fig. 3B), suggesting that HPSE could activate ERK signaling in DN. Therefore, we chose ERK for further signaling study.

### High glucose induced glomerular EndMT through upregulating HPSE and p-ERK1/2

**Fig. 4:**
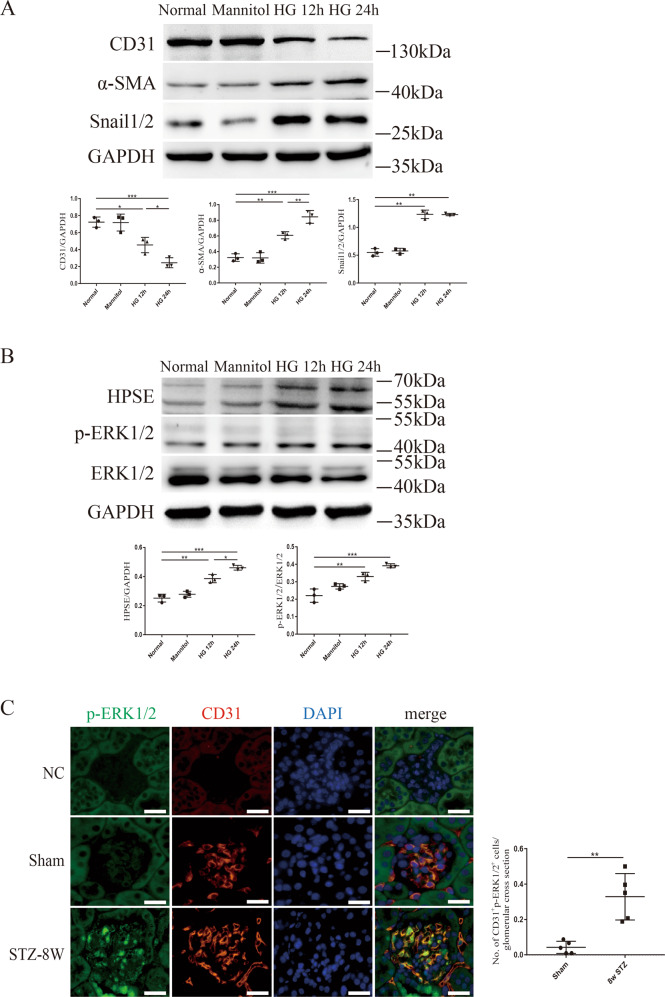
High glucose promoted EndMT in cultured GEnCs through upregulating HPSE and p-ERK1/2. **A** High glucose significantly decreased the protein level of CD31 while increased the expressions of mesenchymal markers including α-SMA and Snail1/2. **B** The protein levels of HPSE and p-ERK1/2 were significantly upregulated by high glucose treatment. **C** The count of double positive IF staining for p-ERK1/2 and CD31 was dramatically increased in STZ-induced diabetic mice, compared with the negative double staining in sham animals. Scale bar = 10 μm. Each vertical bar represents the mean ± SD (*n* = 3) analyzed by one-way analysis of variance (ANOVA) followed by Tukey’s multiple comparisons test. *P < 0.05, **P < 0.01, and ***P < 0.001.

In cultured GEnCs, high glucose dramatically down-regulated the expressions of endothelial marker CD31 and up-regulated the expressions of mesenchymal markers including α-SMA and Snail1/2 at 12 h and 24 h respectively by western blot analysis (Fig. 4A). Simultaneously, high glucose significantly increased the protein levels of HPSE and p-ERK1/2 (Fig. 4B). Mannitol was used as osmotic control and did not show different effects compared with normal glucose. In STZ-induced diabetic mice, double positive IF staining of p-ERK1/2 and CD31 was observed in diabetic glomeruli, while sham animals showed negative staining of p-ERK1/2 in glomeruli (Fig. 4C). The results suggest that high glucose induces GEnCs undergoing EndMT via mediating HPSE and p-ERK1/2 signaling pathway.

### HPSE siRNA inhibited high glucose-induced EndMT in GEnCs through down-regulating p-ERK1/2

**Fig. 5:**
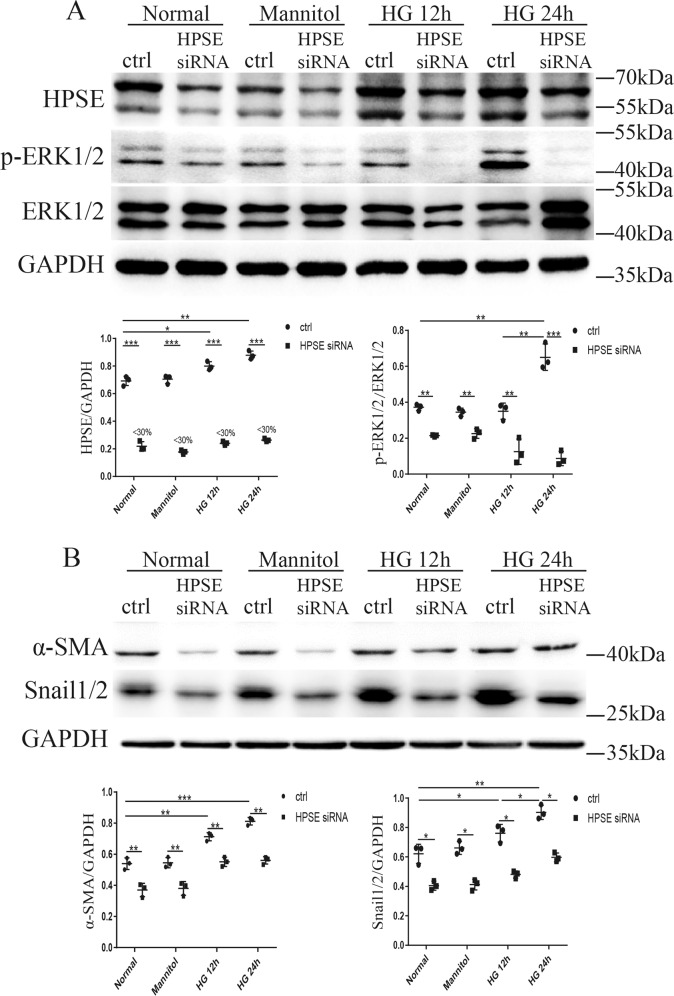
HPSE siRNA inhibited EndMT in cultured GEnCs through inhibiting p-ERK1/2. **A** HPSE siRNA silencing decreased the expression of HPSE by 70%. Compared with normal glucose, high glucose-increased the expressions of p-ERK1/2 (**A**) and mesenchymal markers including α-SMA and Snail1/2 (**B**), both of which were significantly alleviated by HPSE siRNA. Each vertical bar represents the mean ± SD (*n* = 3) analyzed by one-way analysis of variance (ANOVA) followed by Tukey’s multiple comparisons test. *P < 0.05, **P < 0.01, and ***P < 0.001.

HPSE was knocked down in cultured GEnCs by siRNA transfection to study its effects on EndMT in cultured GEnCs. HPSE siRNA silencing decreased the expression of HPSE by 70% (Fig. 5A). Compared with scrambled control siRNA or normal glucose treatment, HPSE siRNA significantly down-regulated p-ERK1/2 expression (Fig. 5A), concomitant with the decreased expressions of mesenchymal markers α-SMA and Snail1/2 which were up-regulated by high glucose treatment (Fig. 5B). Mannitol was used as osmotic control and did not show different effects compared with normal glucose. The results suggest that HPSE and p-ERK1/2 play essential roles in high glucose-mediated EndMT of GEnCs.

### Recombinant mouse HPSE (rmHPSE) induced EndMT of GEnCs through up-regulating p-ERK1/2 in a dosage- and time-dependent manner

**Fig. 6:**
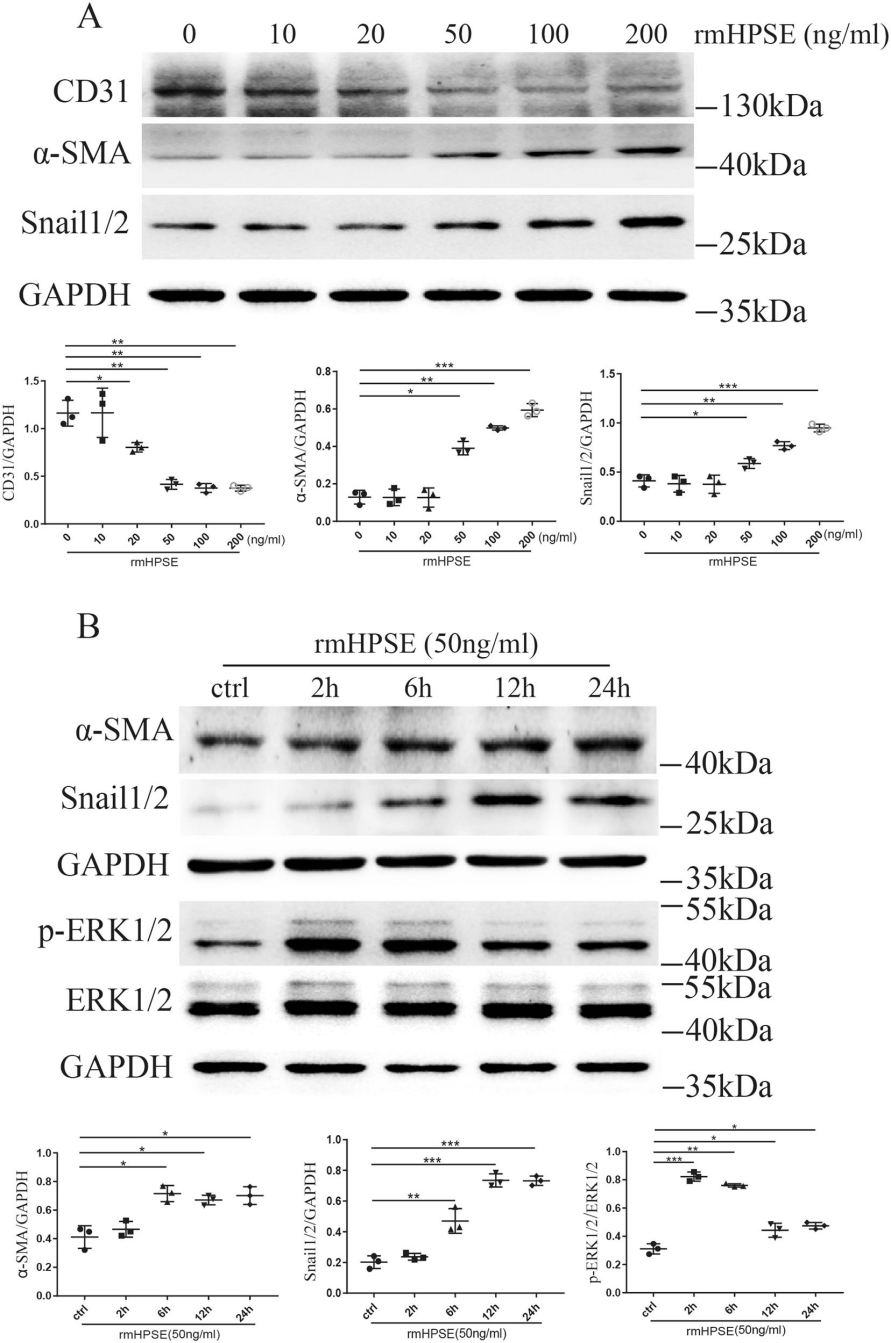
rmHPSE induced EndMT in cultured GEnCs. **A** rmHPSE downregulated the protein level of CD31 and upregulated the expressions of α-SMA and Snail1/2 in a dosage-dependent manner. **B** At the concentration of 50 ng/ml, rmHPSE increased the expressions of p-ERK1/2 and the mesenchymal markers including α-SMA and Snail1/2 in a time-dependent manner. Each vertical bar represents the mean ± SD (*n* = 3) analyzed by one-way analysis of variance (ANOVA) followed by Tukey’s multiple comparisons test. *P < 0.05, **P < 0.01, and ***P < 0.001.

We treated the cultured GEnCs in normal glucose medium with rmHPSE at different concentrations from 0 to 200 ng/ml for 24 h. With the increase of rmHPSE concentration, the expression of CD31 gradually decreased while those of mesenchymal markers including α-SMA and Snail1/2 gradually increased (Fig. 6A). At the concentration of 20 ng/ml, CD31 was significantly decreased by rmHPSE stimulation. At 50 ng/ml, rmHPSE markedly increased the protein levels of α-SMA and Snail1/2. Then, we treated cells with 50 ng/mL rmHPSE for different time periods from 2 h to 24 h (Fig. 6B). p-ERK1/2 was dramatically up-regulated as early as 2 h. Meanwhile, α-SMA and Snail1/2 were significantly increased from the time point of 6 h. The results suggest that HPSE itself could directly induce EndMT of GEnCs through up-regulating p-ERK1/2, suggesting a pivotal role of HPSE in EndMT of diabetic GEnCs.

### p-ERK1/2 was imperative for high glucose or HPSE-mediated EndMT of GEnCs

**Fig. 7:**
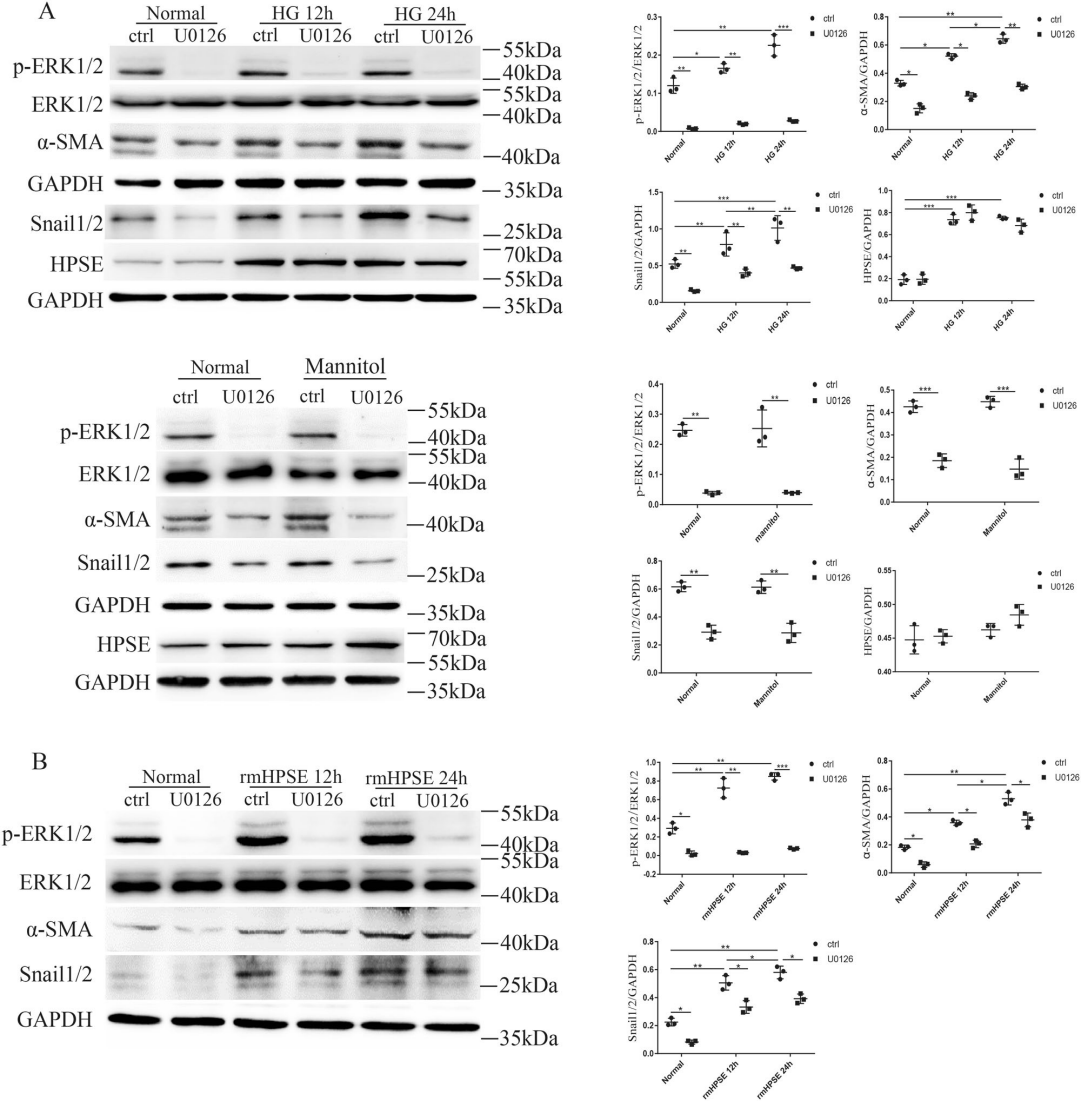
p-ERK1/2 was a vital signaling molecule in HPSE-mediated diabetic glomerular EndMT. **A** Under normal glucose or high glucose conditions, U0126, a specific MEK1/2 inhibitor, dramatically inhibited the expressions of p-ERK1/2 and the mesenchymal markers including α-SMA and Snail1/2, without affecting HPSE protein levels under the same treatments. Mannitol as osmotic control did not show different effects. **B** U0126 markedly blocked rmHPSE-induced expressions of p-ERK1/2, α-SMA and Snail1/2 at 12 h and 24 h, respectively. Each vertical bar represents the mean ± SD (*n* = 3) analyzed by one-way analysis of variance (ANOVA) followed by Tukey’s multiple comparisons test. *P < 0.05, **P < 0.01, and ***P < 0.001.

We pretreated cells with a specific MEK1/2 inhibitor U0126 before high glucose treatment or rmHPSE stimulation. U0126 dramatically inhibited the expressions of p-ERK1/2 as well as mesenchymal markers either under normal glucose or high glucose conditions, without affecting HPSE protein levels under the same experimental conditions (Fig. 7A). Mannitol was used as osmotic control and did not show significant effects compared with normal glucose. Furthermore, U0126 significantly blocked rmHPSE-mediated effects on the above molecules (Fig. 7B). These data suggest that p-ERK1/2 is an imperative downstream signaling molecule in high glucose or HPSE-mediated EndMT of diabetic GEnCs.

## Discussion

EndMT is a process similar to the better understood epithelial-mesenchymal transition (EMT), in which endothelial cells show an acquired mesenchymal characteristics and loss of endothelial features. This phenotypic change has been implicated in development and several pathologic conditions such as cancer, transplant, atherosclerosis and organ fibrosis [3, 11]. In the kidney field, EndMT has been proved to contribute to renal interstitial fibrosis [23, 24]. By endothelial lineage tracing, Zeisberg et al. [25] and Li et al. [26] confirmed the presence of EndMT-derived fibroblasts and myofibroblasts in three mouse models of chronic kidney disease including STZ-induced DN mice, unilateral ureteral obstructive nephropathy and Alport renal disease. What’s more, GEnCs also underwent EndMT in STZ-induced diabetic rats [14] and diabetic db/db mice [15] as well as in the patients with DN [15, 17]. In cultured GEnCs, a few treatments including high glucose [13, 15, 17], advanced glycation end products (AGEs) [16], transforming growth factor (TGF)-β2 [14] and advanced oxidation protein products [27] triggered the process of EndMT. Additionally, AGEs induced EndMT in STZ-induced diabetic mice [16]. Although the mesenchymal-like phenotype helps GEnCs escape from death or apoptosis, these data suggest that EndMT of GEnCs maybe a novel pathway leading to glomerulosclerosis and thus the development and progression of DN.

Being the sole mammalian endoglycosidase, HPSE not only exerts a unique enzymatic degradation of heparan sulfate (HS), thereby facilitating the development of albuminuria and further glomerular diseases, but also plays several non-enzymatic functions in kidney diseases [5–7]. HPSE is recently shown to be involved in mesenchymal transition. HPSE promoted EMT of renal tubular cells either following ischemia/reperfusion injury [28, 29] or during DN [30–32], through regulating pro-fibrotic growth factors including TGF-β [31, 32] and fibroblast growth factors −2 [30, 33]. Specific HPSE inhibition reversed high glucose-induced mesothelial-to-mesenchymal transition, suggesting a promising therapeutic tool to minimize fibrosis in patient on peritoneal dialysis [34]. These data indicate a positive correlation between HPSE and mesenchymal transition.

Glomerular HPSE expression is increased in most proteinuric diseases [5–7]. Diabetes is one of the strongest inducers of HPSE expression [5]. In experimental DN models, overexpressed HPSE was correlated with the extent of albuminuria and renal damage [7–9, 35], which were markedly alleviated by the specific HPSE inhibitor SST0001 [8]. In addition, the deletion of HPSE gene protected diabetic mice from DN [8]. Consistent with the previous reports, the present study also showed that the overexpressed glomerular HPSE had a positive correlation with proteinuria and renal histopathology in STZ-induced diabetic mice, suggesting a remarkable role of HPSE in diabetic glomerulopathy. Moreover, we applied double IF staining and found that partial HPSE was located in diabetic GEnCs which expressed mesenchymal markers α-SMA and Snail1/2. Further correlation analysis revealed that glomerular HPSE expression had a positive correlation with the number of GEnCs undergoing EndMT. In the cultured GEnCs, high glucose significantly upregulated HPSE and the mesenchymal markers, which were dramatically blocked by HSPE siRNA. Intriguingly, rmHPSE treatment dramatically promoted EndMT of cultured GEnCs. These data suggest a distinct role of HPSE in the process of EndMT in diabetic GEnCs, and would expand our knowledge about the multifunctional mechanisms of HPSE in DN development. To our knowledge, this is the first report about the association between HPSE and EndMT of diabetic GEnCs.

ERK belongs to the family of mitogen-activated protein kinases (MAPK) and regulates diverse physiological and pathophysiological processes, such as gene expression, cell division, survival, apoptosis, differentiation, and motility function [19, 36]. Among the six identified members of the ERK family, ERK1/2 are the two most studied kinases and the main regulators of the MAPK/ERK signal pathway. Once the upstream MEK1/2 phosphorylates ERK1/2, it translocates to the nucleus to phosphorylate different targets. ERK phosphorylation contributes to DN development and may be distinctly involved in diabetic glomerular lesions [21]. In patients with DN, the number of glomerular p-ERK1/2-positive cells increased in accordance with the progression of glomerular lesions [21]. Activation of ERK1/2 was also observed in cultured GEnCs treated by high glucose [17, 22] as well as in the lysates of isolated glomeruli and cortices obtained from STZ-induced diabetic mice [37]. In AGEs-treated GEnCs, the co-cultured M2 macrophages may protect GEnCs from damage via inhibiting p-ERK1/2 expression [20]. Our bioinformatics analysis revealed a positive correlation between HPSE and ERK2 in the patients with DN. By in situ double immunostaining analysis, we observed a positive expression of p-ERK1/2 in GEnCs of STZ-induced diabetic mice. Furthermore, the inhibition of ERK1/2 by U0126, a specific MEK1/2 inhibitor, markedly attenuated either high glucose- or rmHPSE-induced expression of the mesenchymal markers in the cultured GEnCs. These results suggest that HPSE promotes EndMT of diabetic GEnCs via activating ERK signaling.

There are some shortcomings in our manuscript. First, a specific overexpression of HPSE is needed to directly validate its effect on EndMT. Second, in addition to ERK1/2, some key intermediate signaling molecules in HPSE-mediated EndMT are still need to be explored. Third, STZ-induced diabetes mice is an established model for generating type 1 diabetes. Whether and how HPSE regulates glomerular EndMT in type 2 diabetes may help us to further understand the roles of HPSE in the process of EndMT of GEnCs. The related studies are our ongoing investigations.

In summary, glomerular HPSE expression was upregulated in STZ-induced diabetic mice and was positively correlated with proteinuria, renal histopathology and EndMT of GEnCs. In the cultured GEnCs, high glucose upregulated the expressions of HPSE and p-ERK1/2 which were inhibited by HPSE siRNA. rmHPSE promoted mesenchymal transition of GEnCs via activating ERK1/2 signaling. These data reveal a distinct effect of HPSE on the process of EndMT of diabetic GEnCs. A better understanding of the key role of HPSE in EndMT may help to develop more specific therapeutic interventions targeting GEnCs dysfunction in DN.

## Methods and materials

### Mice model of STZ-induced diabetic nephropathy (DN)

All animal procedures were conducted according to the guidelines for the care and use of laboratory animals approved by Department of Laboratory Animal Science, Fudan University (Animal Protocol number 20171509A348). Male DBA/2 J mice were purchased from Shanghai SLAC Laboratory Animal Center (Shanghai, China) at 6–8 weeks of age. After 2 weeks of adaptive feeding and fasting for 12 h (but water), mice were randomly assigned to two groups: sham group (*n* = 4~5 per group), and STZ-treated group (*n* = 5~7 per group). Diabetes was induced by daily intraperitoneal injection of STZ (40 mg/kg BW, S0130: Sigma-Aldrich, St. Louis, USA, made freshly in 0.1 mol/L citrate buffer, pH 4.5) for 5 consecutive days as previously reported [38]. Control mice were injected with an equivalent volume of citrate buffer. Blood glucose levels were determined 7 days after STZ injection, and only mice with fasting blood glucose concentrations >16 mmol/L were used for later experiments. 24 h urine was collected before the mice were sacrificed under sodium pentobarbital anesthesia at 4 and 8 weeks respectively after the onset of diabetes, which were at the early stages of DN [16, 26, 38]. 24 h urinary protein excretion is calculated based on the concentration by using Pierce™ BCA Protein Assay Kit (Lot23227, Thermo Fisher, Massachusetts, USA) and 24 h urine volume. Kidneys were dissected and were fixed in 4% paraformaldehyde solution for histology and immunostaining.

### Renal histopathology

A total of 3 µm sections of kidney tissue were stained with hematoxylin-eosin and periodic acid-Schiff (PAS). PAS-stained kidney sections were used to evaluate glomerular mesangial matrix as previously described [39]. Briefly, photomicrographs of PAS stained sections were taken at a 200x magnification from non-overlapping fields. 30 glomeruli were assessed in each mouse kidney. Glomerular and mesangial areas were respectively determined by manually tracing the tuft perimeter and automatically measuring the PAS positive material in each glomerulus with ImageJ-Pro Plus 6.0. The mesangial matrix index was calculated as the ratio of the mesangial area to the glomerular area × 100 (% area). A whole kidney mesangial matrix index was obtained by averaging indexes from all glomeruli on one section. 4~7 mice per experimental condition were analyzed. All slides were analyzed in a blinded fashion.

### Double immunofluorescence staining and immunohistochemical staining

Double immunofluorescence (IF) staining of the paraffin-embedded kidney sections was performed as previously described [40]. After deparaffinization and rehydration, sections were boiled in citrate or Tris-EDTA buffer for antigen retrieval, blocked for 30 min at 37 °C and then incubated with first primary antibodies [CD31: 7769, 1:5000, Proteintech, California, USA; α-SMA: ab5694, 1:5000, Cambridge, UK; Snail1/2: ab180714, 1:5000, Abcam, Cambridge, UK; HPSE: 1:3000, ab85543, Abcam, Cambridge, UK; phospho-ERK1/2 (p-ERK1/2): 4370, 1:5000, Cell Signaling Technology, Massachusetts, USA] overnight at 4 °C, followed by incubation with Polymer HRP-conjugated mouse or rabbit IgG and with Fluorescent Tyramide Signal Amplification reagents (Opal 4-Color Fluorescent IHC Kit, PerkinElmer, Connecticut, USA) according to the manufacturer’s instructions at room temperature for 10 min in the dark. After washing, the sections were microwaved in citrate or Tris-EDTA buffer solution to strip the primary-secondary-HRP complex allowing introduction of the second primary antibodies for the detection of the next target protein. Ten photomicrographs per section were randomly taken at a 200× magnification (*n* = 5). IF signals were captured on a Leica DMi8 (Wetzler, Germany) fluorescence microscope attached a digital camera. Fluorescence excitation wavelengths were 340–380 nm for DAPI, 450–490 nm for FITC, and 517–563 nm for RHOD, respectively.

Immunohistochemistry (IHC) staining for HPSE was performed on the paraffin-embedded kidney sections. After deparaffinization and rehydration, 3 µm-sections were microwaved in citrate buffer at 900 W for 2.5 min and 150 W for 15 min for antigen retrieval. After blocking for 30 min at 37 °C, the sections were incubated with HPSE antibody (1:100, ab85543, Abcam, Cambridge, UK) overnight at 4 °C, followed by HRP-conjugated-IgG. DAB was used as the chromogenic agent, and hematoxylin was used for nuclei counterstaining. Ten photomicrographs per section were randomly taken at a 200× magnification (*n* = 5). IHC data were analyzed with ImageJ. All slides were analyzed in a blinded fashion. Slides were captured on a Nikon Y-THM (Tokyo, Japan) microscope connected to a digital camera.

### Bioinformatics analysis

We obtained the gene expression profile data of DN patients from the GEO database (GSE14202). Pearson correlation analysis was used to analyze the co-expression relationship between HPSE and related genes. *P* values < 0.05 was considered statistically meaningful. All statistical analyses were performed using R software (3.6.2).

### Cell culture and treatment

Mouse GEnCs were cultured in DMEM medium (01-051-1 A, Biological industries, Kibbutz Beit-Haemek, Israel) containing 10% fetal bovine serum (FBS, Sigma-Aldrich, St. Louis, USA) at 37 °C and 5% CO_2_. After 6 h cultured in DMEM medium without serum, the cells were immediately treated with 25 mM glucose (06-1055-57-1 A, Biological Industries, Kibbutz Beit-Haemek, Israel) or recombinant mouse HPSE (rmHPSE, 9788-GH-005, Novus Biologicals, Colorado, USA) for indicated time periods. Mannitol of the same concentration as high glucose was used as osmolality control. To observe the possible role of p-ERK1/2, the cells were pretreated with U0126 (10 μM, 9903, Cell Signaling Technology, Massachusetts, USA), a specific MEK1/2 (the upstream kinase of ERK1/2) inhibitor, for 1 h before high glucose or rmHPSE treatment.

### Western blot analysis

Total cell lysates were extracted with NP40 lysis buffer (P0013F, Beyotime, Shanghai, China). 15 µg of total protein were electrophoresed with SDS-PAGE and transferred onto PVDF membranes. After blocking with 5% non-fat milk for 1 h, membranes were probed with primary antibodies against target antigens (as mentioned above), or GAPDH (5174 S, 1:1000, Cell Signaling Technology, Massachusetts, USA) as internal controls, at 4 °C overnight. After incubation with HRP-conjugated IgG (1:2000, ab6789, Abcam, Cambridge, UK), blots were developed with enhanced chemiluminescence developing solutions and quantified using ImageJ software.

### Transfection of HPSE siRNA

Silencing of HPSE was performed by transfecting GEnCs with HPSE siRNA or scrambled control siRNAs synthesized by Genomeditech, Shanghai, China. Transfections were performed with TranslT-X2 according to the manufacturer’s instructions (Mirus Bio, Wisconsin, USA). HPSE siRNA Sequences (5’−3’: CCAUGAUAUUUGCAGGUCUAGACCUGCAAAUAUCAUGG).

### Statistical analysis

Quantitative data were representative of at least three times’ experiments. The results were expressed as mean ± standard deviation (SD). ImageJ software was used to measure the gray value of the Western Blot analysis, and protein relative density was represented by the ratio of target protein gray value/GAPDH gray value. Integrated Density and area for IF and optical density for PAS staining were analyzed with the ImageJ-Pro Plus software. All the results were analyzed by GraphPad Prism 7 software. Gaussian distribution of the data was verified using the D’Agostino & Pearson omnibus normality test. Data between two groups were analyzed by independent sample two-tailed t-tests and the differences among groups were analyzed by one-way ANOVA or two-way ANOVA followed by Tukey’s multiple comparisons test. According to the distribution of sample, Pearson correlation coefficients were used for correlation analysis. The correlation is negligible when correlation coefficients |R | is below 0.1, small when |R | ranges from 0.1 to 0.3, moderate when |R | ranges from 0.3 to 0.5, and large when |R | is >0.5. *P* values < 0.05 were considered statistically significant.

## Supplementary information

Original figures

## Data Availability

The raw data supporting the conclusions of this article will be made available by the authors without undue reservation.
