# Correction: Heparanase promotes endothelial-to-mesenchymal transition in diabetic glomerular endothelial cells through mediating ERK signaling

**DOI:** 10.1038/s41420-022-00907-8

**Published:** 2022-03-09

**Authors:** Kaili Chang, Qiyuan Xie, Jianying Niu, Yong Gu, Zhonghua Zhao, Fengxia Li, Qiaojing Qin, Xueguang Liu

**Affiliations:** 1 Department of Pathology, School of Basic Medical Sciences, Fudan University, 131 Dongan Road, 200032 Shanghai, China; 2 Department of Nephrology, Zhongshan Hospital, Fudan University, 180 Fenglin Road, 200032 Shanghai, China; 3 Department of Nephrology, The Fifth People’s Hospital, Fudan University, 801 Heqing Road, 200240 Shanghai, China; 4 Center of Community-Based Health Research, Fudan University, 801 Heqing Road, 200240 Shanghai, China; 5 Department of Nephrology, Huashan Hospital, Fudan University, 12 Middle Urumqi Road, 200040 Shanghai, China

**Keywords:** Experimental models of disease, Diabetic nephropathy

Correction to: *Cell* *Death Discovery* 10.1038/s41420-022-00858-0, published online 16 February 2022

The original version of this article unfortunately contained a mistake in the affiliations. The original article has been corrected.

