# Supplementary material for: Heparanase promotes endothelial-to-mesenchymal transition in diabetic glomerular endothelial cells through mediating ERK signaling
Source: Cell Death Discov. 2022 Feb 16;8:67. doi: 10.1038/s41420-022-00858-0 (PMC8850459; doi:10.1038/s41420-022-00858-0)
Supplement: Supplementary file 1 — Original figures [file 41420_2022_858_MOESM1_ESM.pdf]

Fig 4

A

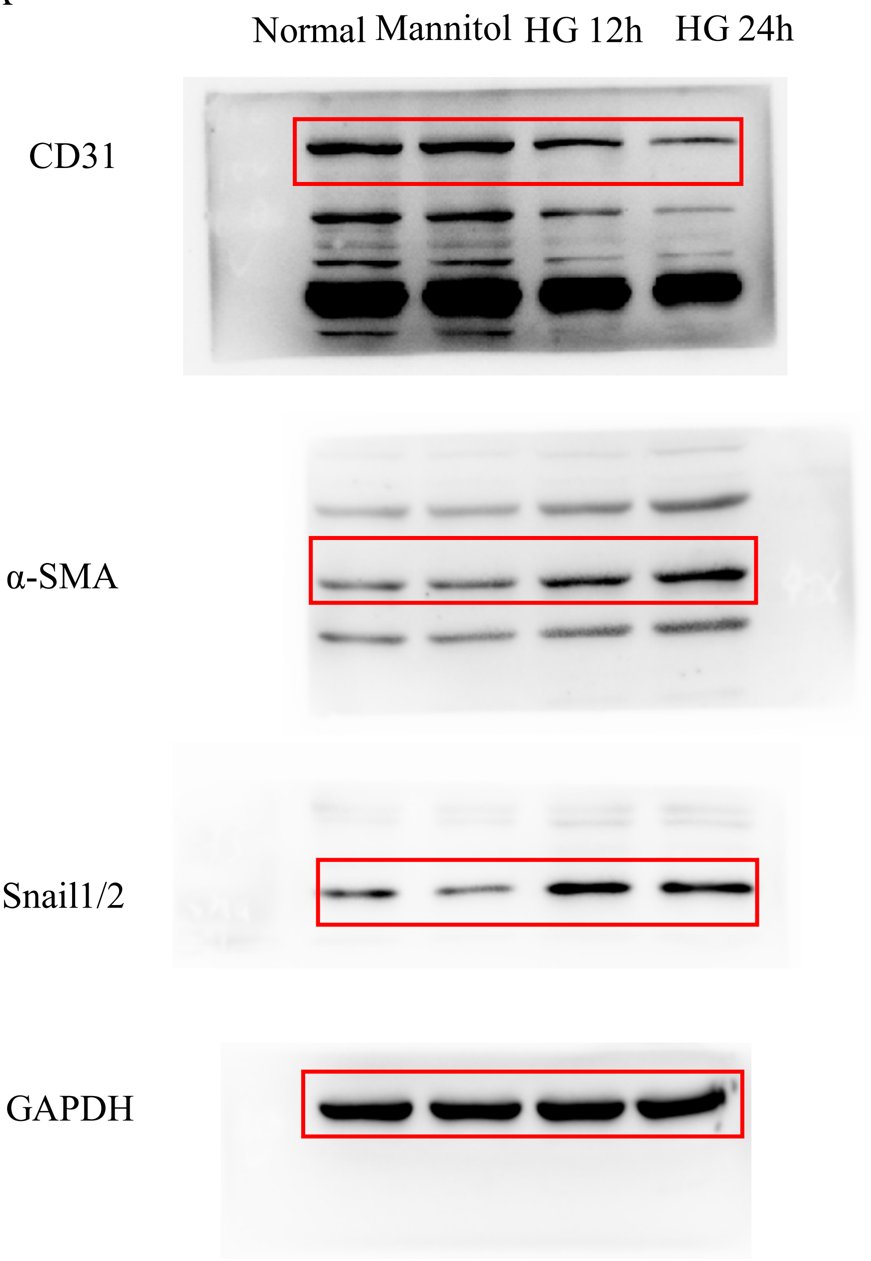

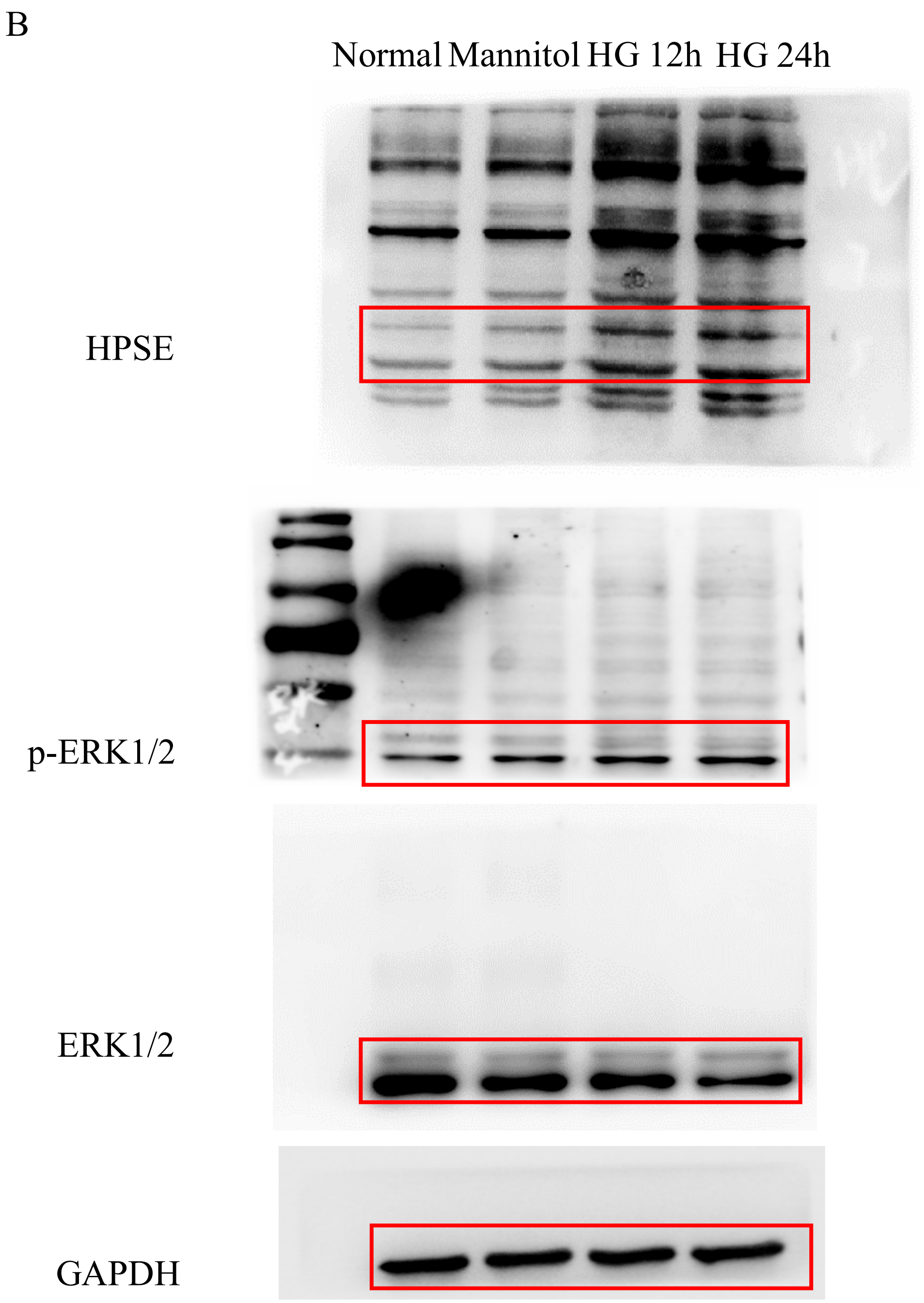
B
Normal Mannitol HG 12h HG 24h
HPSE
p-ERK1/2
ERK1/2
GAPDH

Fig 5

A

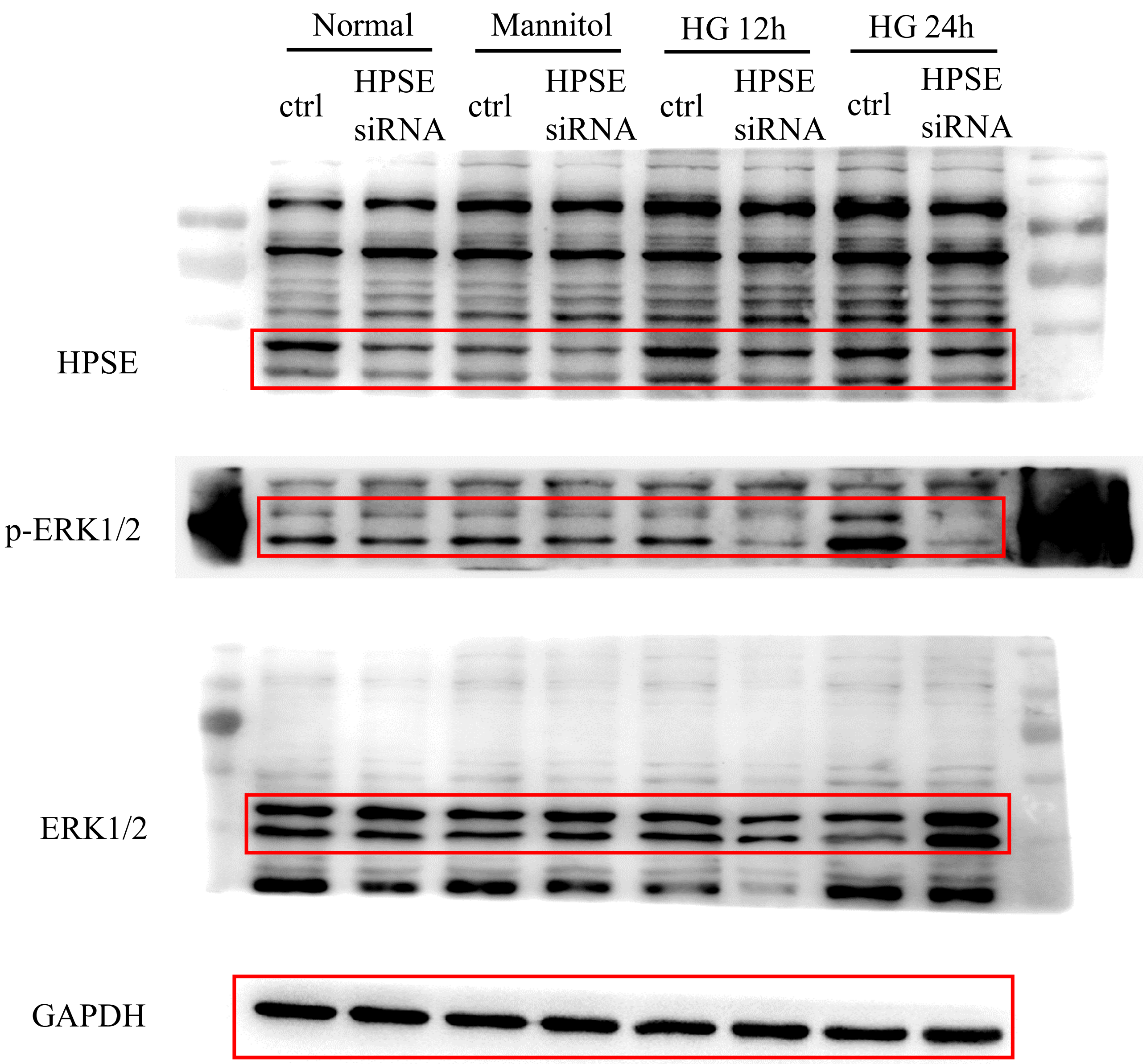
Normal
Mannitol
HG 12h
HG 24h
ctrl
HPSE siRNA
ctrl
HPSE siRNA
ctrl
HPSE siRNA
ctrl
HPSE siRNA
HPSE
p-ERK1/2
ERK1/2
GAPDH

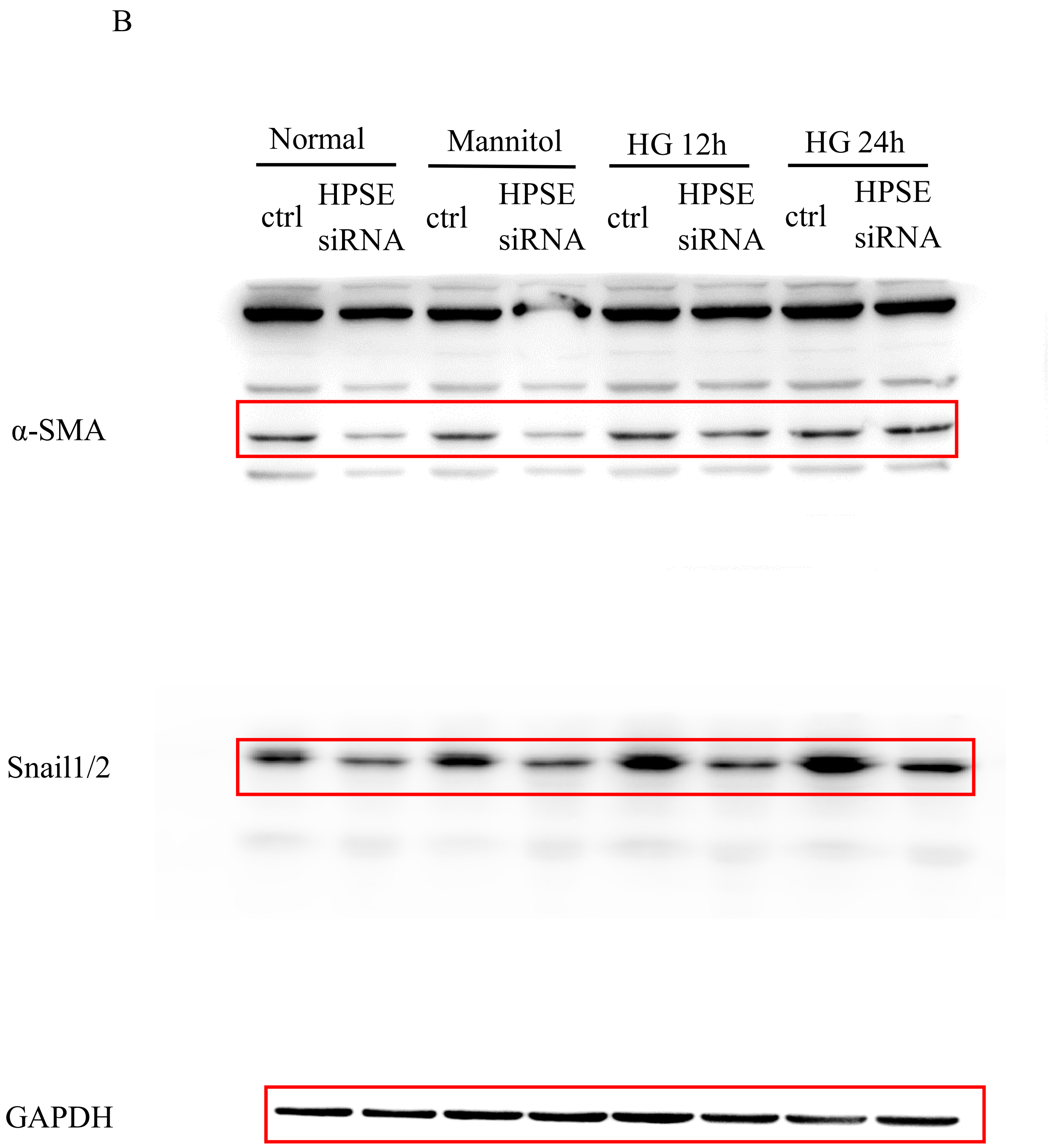
B
Normal
Mannitol
HG 12h
HG 24h
ctrl
HPSE siRNA
ctrl
HPSE siRNA
ctrl
HPSE siRNA
ctrl
HPSE siRNA
α-SMA
Snail1/2
GAPDH

Fig 6

A

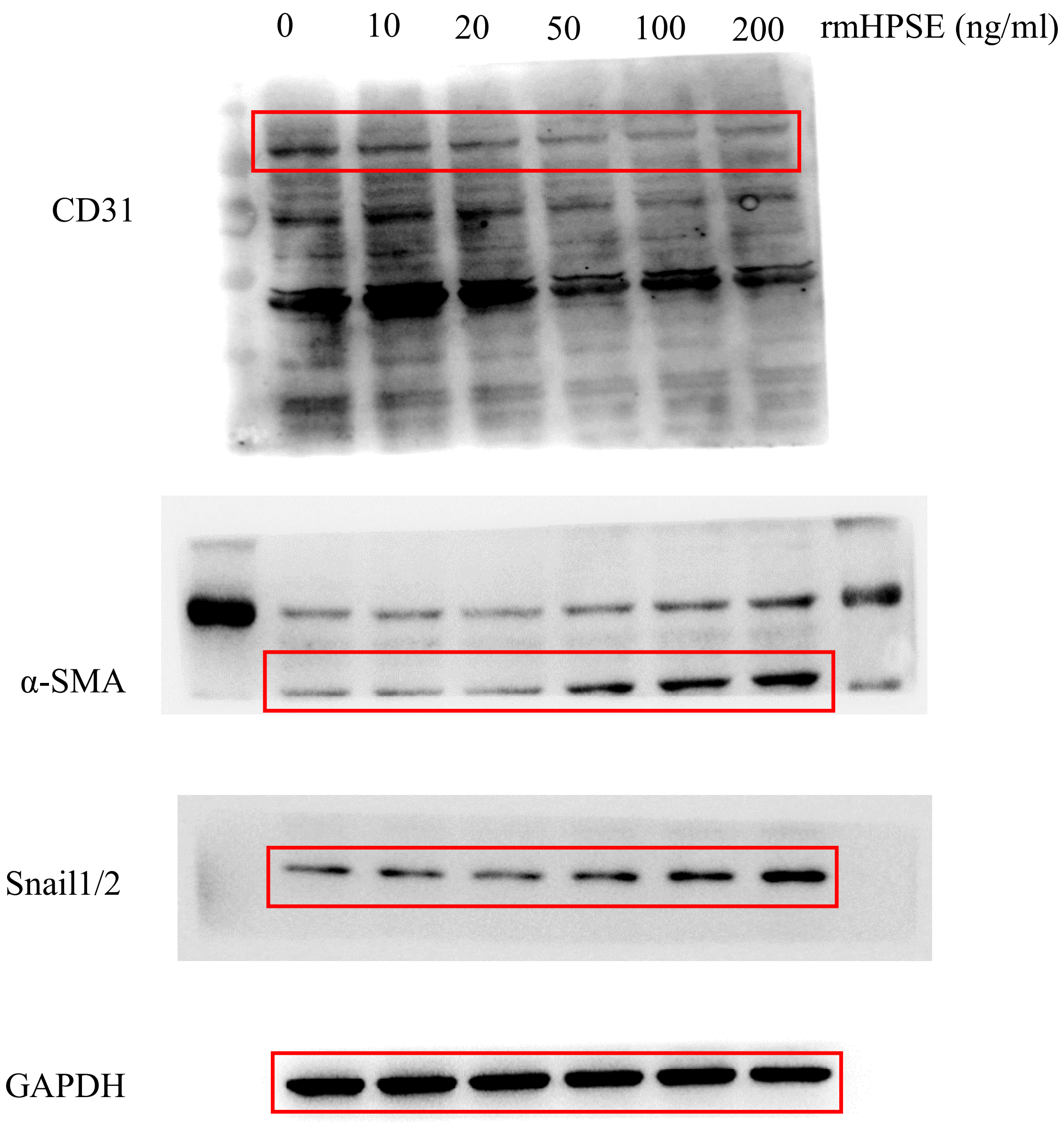

B

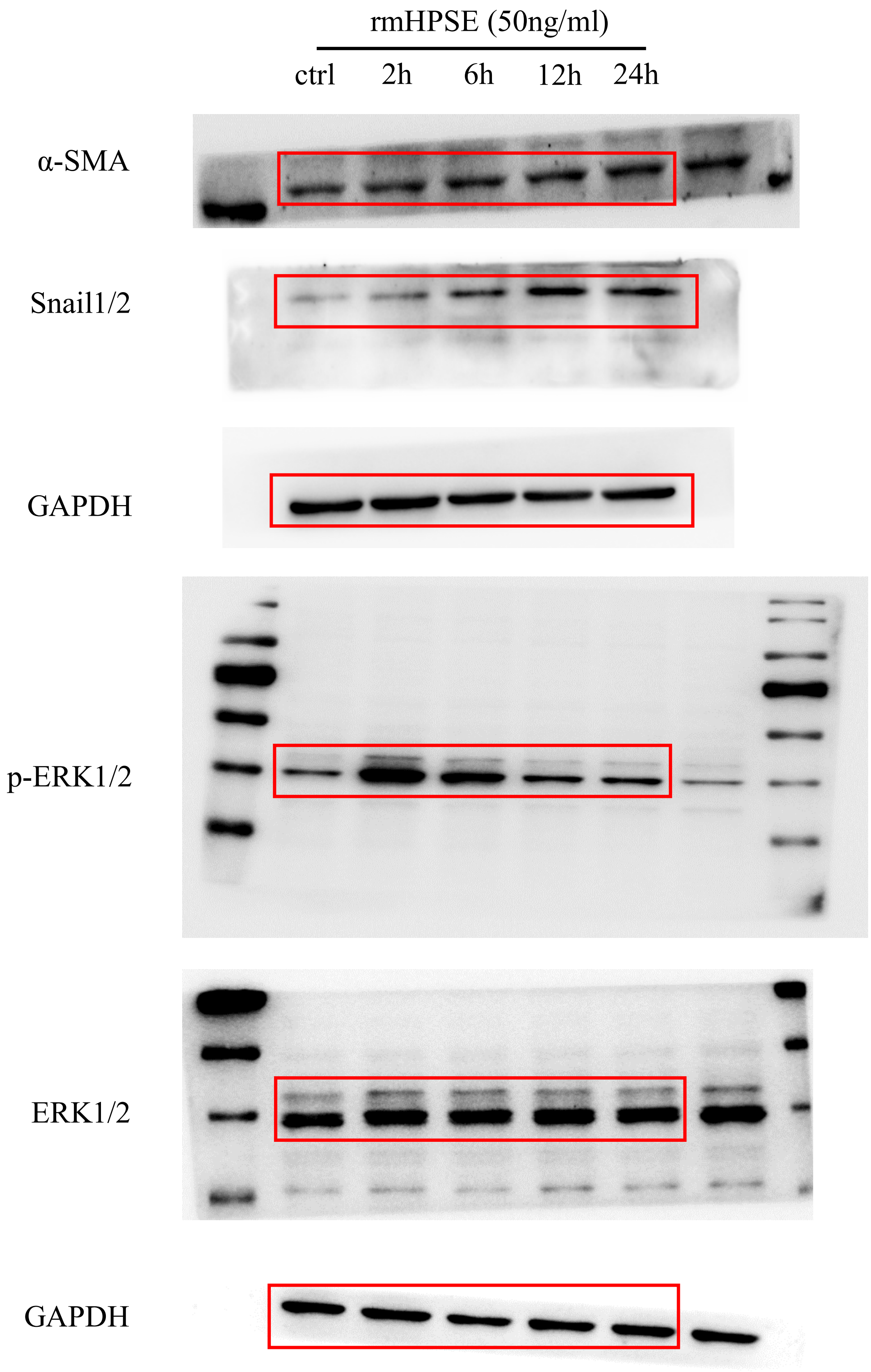

rmHPSE (50ng/ml)
ctrl
2h
6h
12h
24h
α-SMA
Snail1/2
GAPDH
p-ERK1/2
ERK1/2
GAPDH

Fig 7

A

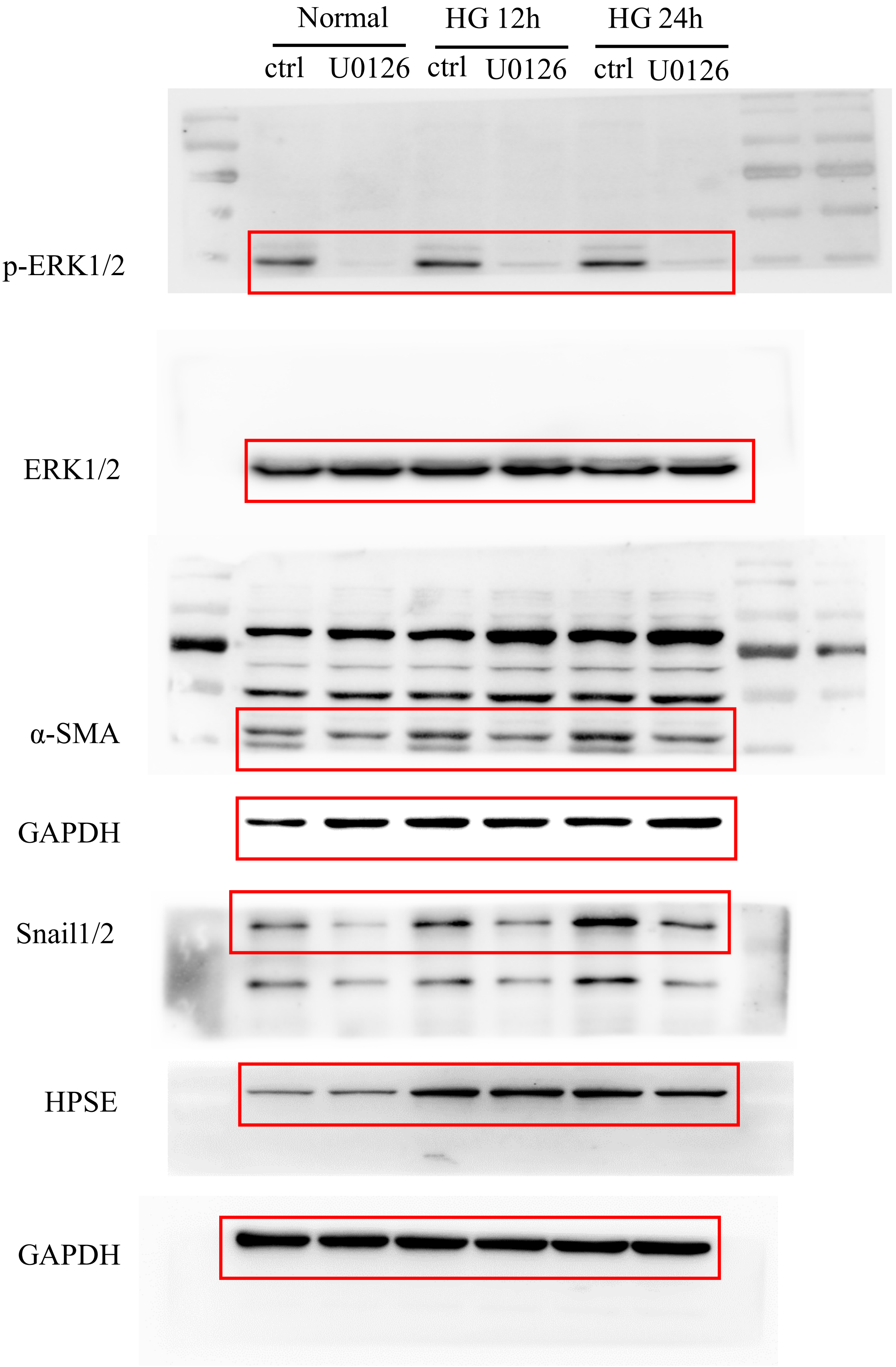
Normal
HG 12h
HG 24h
ctrl
U0126
ctrl
U0126
ctrl
U0126
p-ERK1/2
ERK1/2
α-SMA
GAPDH
Snail1/2
HPSE
GAPDH

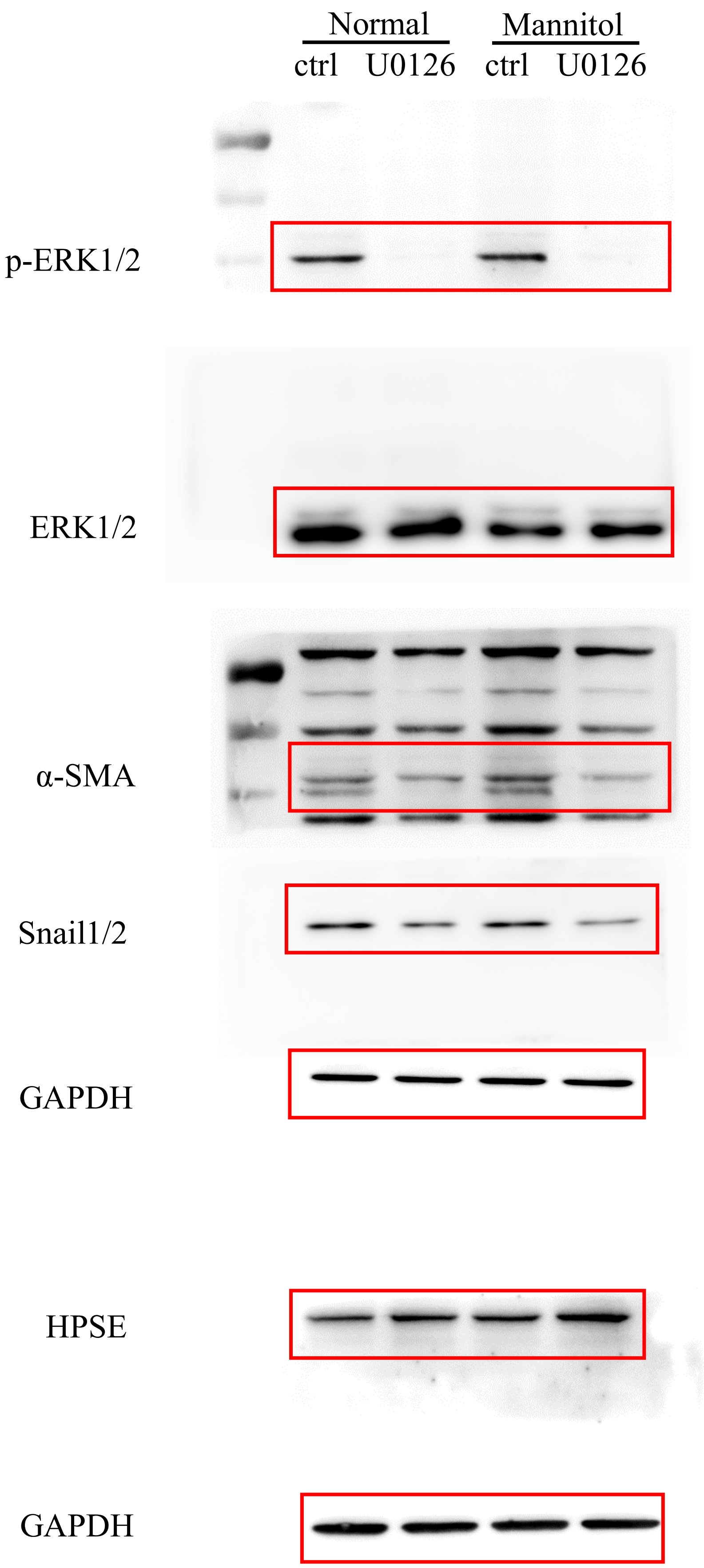
Normal
Mannitol
ctrl
U0126
ctrl
U0126
p-ERK1/2
ERK1/2
α-SMA
Snail1/2
GAPDH
HPSE
GAPDH

B

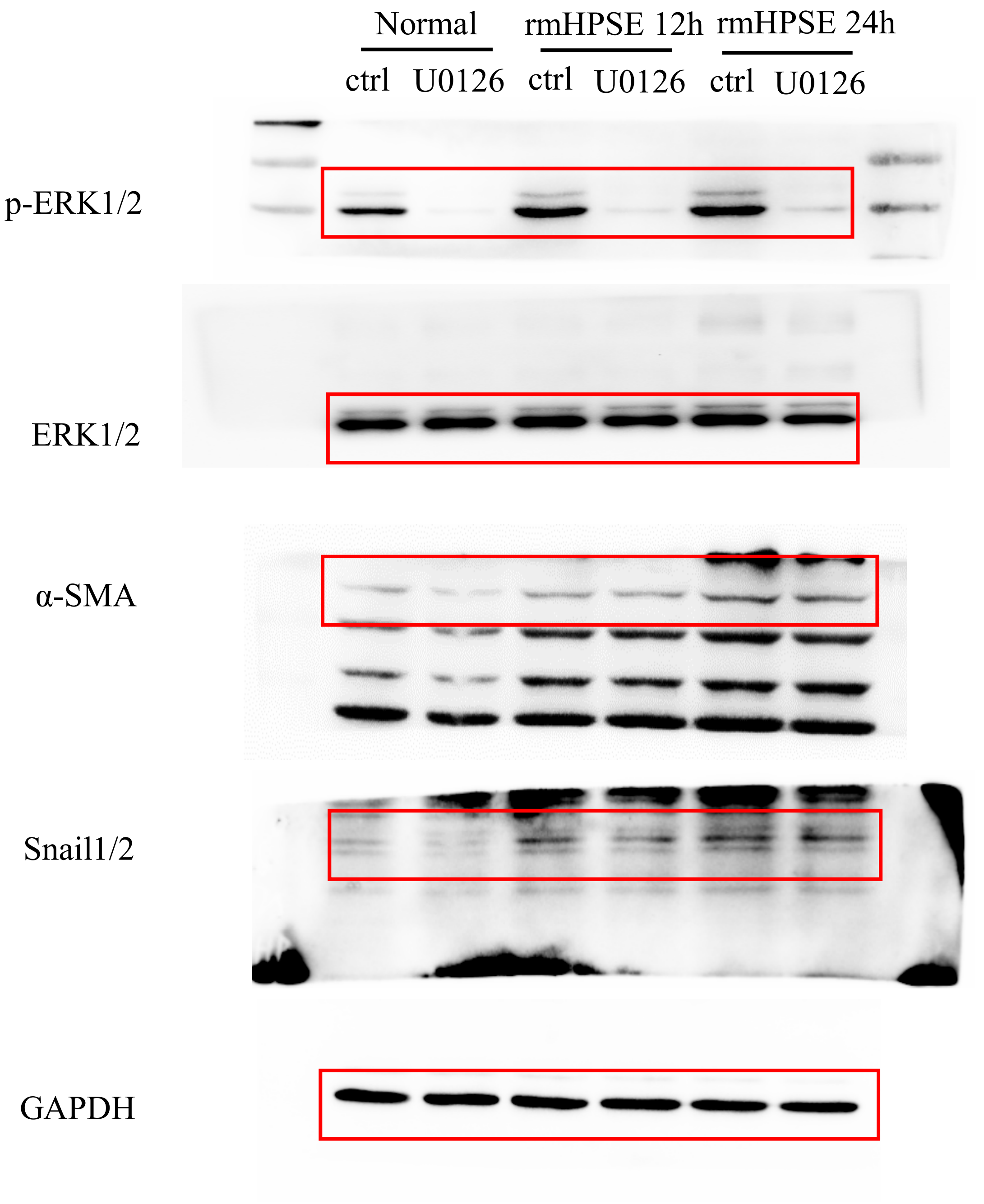
Normal
rmHPSE 12h
rmHPSE 24h
ctrl
U0126
ctrl
U0126
ctrl
U0126
p-ERK1/2
ERK1/2
α-SMA
Snail1/2
GAPDH
